# Redox‐responsive dual‐drug nanomedicine integrating cisplatin and trypsin for synergistic reversal of tumor chemoresistance

**DOI:** 10.1002/smo2.70055

**Published:** 2026-04-23

**Authors:** Xiaolan Yin, Qi Wang, Ming Zhang, Cheng Zhang, Qixian Chen, Haidong Li, Yan Zhao, Yue Wang, Jingyun Wang, Liuwei Zhang, Hongyan Cui

**Affiliations:** ^1^ School of Bioengineering Dalian University of Technology Dalian China; ^2^ Innovation Center of Yangtze River Delta Zhejiang University Jiaxing China; ^3^ Provincial Key Laboratory of Interdisciplinary Medical Engineering for Gastrointestinal Carcinoma Liaoning Cancer Hospital & Institute Shenyang China; ^4^ Department of Gastric Surgery Cancer Hospital of Dalian University of Technology Shenyang China; ^5^ Department of Gastric Surgery Cancer Hospital of China Medical University Shenyang China; ^6^ Provincial Key Laboratory of Interdisciplinary Medical Engineering for Gastrointestinal Carcinoma Liaoning Cancer Hospital & Institute Shenyang China

**Keywords:** cisplatin, protein prodrug, reversal of tumor resistance, tumor targeting

## Abstract

To overcome cisplatin resistance without increasing systemic toxicity, we rationally elaborated a glutathione‐activatable prodrug nanomedicine that chemically co‐encapsulates cisplatin and a masked protease. First, a redox‐labile succinimide linker (NC‐ss‐COOH) was covalently grafted onto the ε‐amino groups of trypsin to create a “pro‐protease” (ssTrypsin) whose catalytic activity is completely silenced in circulation but instantly restored (≥96%) upon cleavage by intratumoral GSH. Simultaneously, cisplatin was stably coordinated to the carboxyl‐rich backbone of cRGD‐PEG‐polyglutamic acid and carboxyl‐rich ssTrypsin, forming a polymer–Pt(II) prodrug that prevents premature Pt‐GSH adduct formation. These two prodrugs co‐self‐assemble into potent anti‐tumor nanoconstructs that actively target α_v_β_3_/α_v_β_5_‐overexpressing tumors. Upon GSH‐triggered activation, the dual‐drug combination exerts complementary actions: (i) released cisplatin directly damages DNA, while (ii) reactivated trypsin proteolytically degrades all intracellular, cytomembrane, and extracellular proteins as possible (including DNA‐repair proteins), collectively re‐sensitizing resistant cells. This “prodrug + pro‐enzyme” strategy yields a 2.5‐fold reduction in IC_50_ against A2780DDP cells and 77% tumor suppression in vivo, all with minimal off‐target toxicity.

## Graphical abstract

This study presents a novel glutathione‐activatable prodrug nanomedicine (cRGD‐PEG‐PGlu‐NP (ssTrypsin & CDDP)) that co‐encapsulates cisplatin and a masked protease to combat cisplatin resistance. The dual‐drug system selectively targets resistant tumor cells, enhancing the efficacy of cisplatin while minimizing systemic toxicity. The results show a significant reduction in IC_50_ values and impressive tumor suppression in vivo, demonstrating a promising strategy for overcoming drug resistance.

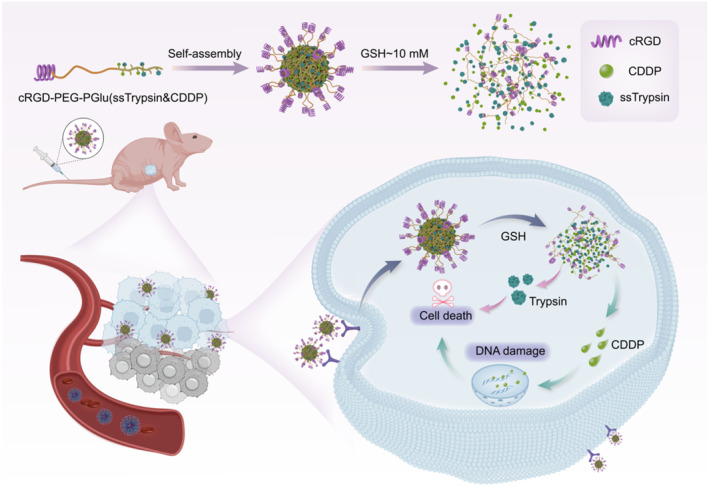

## 1 INTRODUCTION

Platinum‐based chemotherapy remains the cornerstone for ovarian, lung and head‐and‐neck malignancies^[1]^; yet cisplatin's clinical impact is constrained by two immutable barriers: dose‐limiting nephro‐/neurotoxicity and the rapid emergence of glutathione (GSH)‐mediated resistance.[^2^, ^3^, ^4^, ^5^, ^6^, ^7^] Up to 60% of initially responding patients ultimately relapse as cancer cells exploit intracellular GSH to scavenge Pt(II), up‐regulate DNA‐repair enzymes and reinforce their extracellular matrix (ECM), thereby erecting a multi‐layered fortress against further platinum assault.[^8^, ^9^, ^10^, ^11^, ^12^, ^13^, ^14^]

Trypsin, a serine protease, uniquely remodels the tumor microenvironment by hydrolyzing ECM constituents such as fibronectin and collagen.[^15^, ^16^] This proteolysis lowers interstitial pressure, augment nanoparticle penetration and may modulate oncogenic signaling to amplify chemotherapy efficacy.[^17^, ^18^, ^19^] Nevertheless, both protein and platinum therapeutics suffer from poor tumor enrichment and premature inactivation.

Nanomedicine offers a technological counter‐strategy. Nanoparticles (50–150 nm) exploit the enhanced permeability and retention (EPR) effect for passive accumulation, while surface functionalization (e.g., ligands or stimuli‐responsive linkers) confers active targeting and controlled release, simultaneously improving solubility, stability and safety.[^20^, ^21^, ^22^, ^23^, ^24^, ^25^, ^26^, ^27^, ^28^] Representative systems such as the polymeric micelle NC‐6004 reduce cisplatin nephrotoxicity[^29^, ^30^] and liposomal formulations targeting DNA‐repair proteins partially restore platinum sensitivity.^[31]^ However, these approaches intervene in only one resistance axis; the multifactorial nature of tumor defense demands concurrent targeting of GSH detoxification, ECM barrier and DNA‐repair machinery.[^32^, ^33^]

**SCHEME 1:**
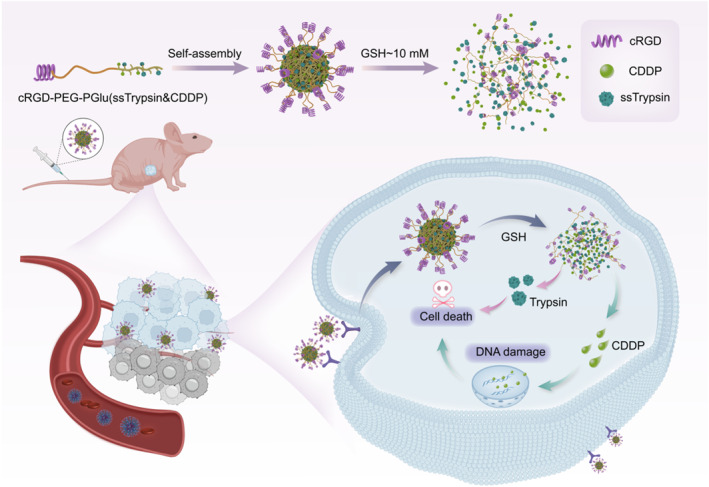
Schematic illustration of the synchronized co‐coordination of cisplatin (CDDP) to a carboxylated trypsin prodrug and to a multifunctional cRGD‐PEG‐PGlu block copolymer, driving the spontaneous self‐assembly of a dual oncologic nanomedicine (ssTrypsin&CDDP). The cyclic RGD ligand confers α_v_β_3_/α_v_β_5_ integrin‐targeted delivery to neovasculature‐rich tumor tissues. Upon cellular internalization, the disulfide tether of the trypsin prodrug is selectively cleaved by the glutathione (GSH)‐abundant intracellular milieu, triggering on‐site activation and burst release of catalytically active trypsin while concurrently depleting GSH. The attenuated antioxidant capacity compromises cisplatin detoxification, amplifying platinum‐induced genotoxicity. Concomitantly, the liberated trypsin executes promiscuous proteolysis of essential intracellular proteins—including DNA‐repair machinery—thereby potentiating a synergistic, multi‐pronged antitumor cascade.

Here we introduce cRGD‐PEG‐PGlu‐NP(ssTrypsin&CDDP), a redox‐activatable nanomedicine that integrates these objectives into a single nanomedicine. A PEG‐poly (glutamic acid) block copolymer self‐assembles with cisplatin via γ‐carboxylate–Pt(II) coordination while electrostatically incorporating disulfide‐caged, carboxylated trypsin. Cyclic RGD mediates integrin α_v_β_3_/α_v_β_5_‐directed tumor homing; high intracellular GSH triggers disulfide cleavage, depleting antioxidant pools and releasing native trypsin. Concomitant ECM degradation and proteolytic demolition of DNA‐repair proteins sensitize cancer cells to platinum‐induced genotoxicity without compromising normal tissue (Scheme 1). This orthogonal, multi‐target strategy yields a 77% tumor growth inhibition in cisplatin‐resistant ovarian xenografts with negligible systemic toxicity, offering an immediately translatable paradigm to overcome clinical platinum resistance.

## 2 RESULTS AND DISCUSSION

### 2.1 Synthesis of multifunctional polyanionic block copolymeric cRGD‐PEG‐PGlu

Leveraging the well‐established coordination between cisplatin and carboxylate moieties, we designed a tumor‐targeted nanomedicine by surface‐decoration of polyethylene glycol‐b‐poly (l‐glutamic acid) (PEG‐PGlu) with the cyclic peptide ligand c(RGDfK). The RGD motif confers high‐affinity binding to integrins α_v_β_3_ and α_v_β_5_, which are markedly up‐regulated on multiple solid tumors, thereby promoting selective accumulation within neoplastic tissue. The complete synthetic route of cRGD‐PEG‐PGlu is depicted in Schemes S3–S4.

Synthesis was started with the chemoselective conjugation of c(RGDfK) to heterobifunctional BOC‐NH‐PEG‐NHS, yielding BOC‐protected cRGD‐PEG‐NH‐BOC. ^1^H‐NMR (Figure S7) confirmed the reaction: the –(CH_2_CH_2_O)– repeat units of PEG resonated at δ 3.5 ppm, whereas the phenyl protons of the c(RGDfK) side‐chain appeared at δ 7.0–7.3 ppm. Integration of these signals results in an average c(RGDfK)/PEG stoichiometry of 0.84. Subsequent treatment with 0.1 M HCl (aq) quantitatively removed the BOC group, as evidenced by the complete disappearance of the tert‐butyl singlet at δ 1.5 ppm (Figure S8), affording cRGD‐PEG‐NH_2_.

The macroinitiator was then employed to ring‐open l‐Glu(OBzl)‐NCA. Polymerization proceeded under rigorously anhydrous conditions, and the resulting cRGD‐PEG‐PGlu(OBzl) was subjected to 1 M NaOH/MeOH to cleave the benzyl esters. ^1^H‐NMR (Figure S9) of the protected intermediate revealed: (i) PEG –(CH_2_CH_2_O)– at δ 4.2 ppm, (ii) PGlu backbone protons at δ 4.8–5.1 ppm, and (iii) benzylic –CH_2_C_6_H_5_ protons at δ 5.2–5.6 ppm. Peak‐area analysis indicated a mean degree of polymerization of 57 for the glutamate block. After deprotection, the benzylic signals vanished entirely (Figure S10), corroborating the quantitative conversion to cRGD‐PEG‐PGlu. A non‐targeted PEG‐PGlu analog was prepared analogously (Scheme S5, Figures S11 and S12) to serve as a control. Collectively, the spectroscopic data confirm the successful and reproducible synthesis of both cRGD‐PEG‐PGlu and PEG‐PGlu constructs.

### 2.2 Synthesis of carboxylated trypsin of prodrug

The key functional building block was prepared in two high‐yielding organic steps (Schemes S1 and S2). Firstly, acylation of the parent scaffold furnished the hydroxyl‐terminated intermediate NC‐ss‐OH. Subsequent Steglich‐type esterification with succinic anhydride afforded the carboxylic acid‐terminated derivative NC‐ss‐COOH. The structures of both intermediates and the final product were fully corroborated by ^1^H‐NMR, ^13^C‐NMR and HRMS; all resonances and exact‐mass data were in excellent agreement with theoretical values (Figures S1–S6).

**FIGURE 1:**
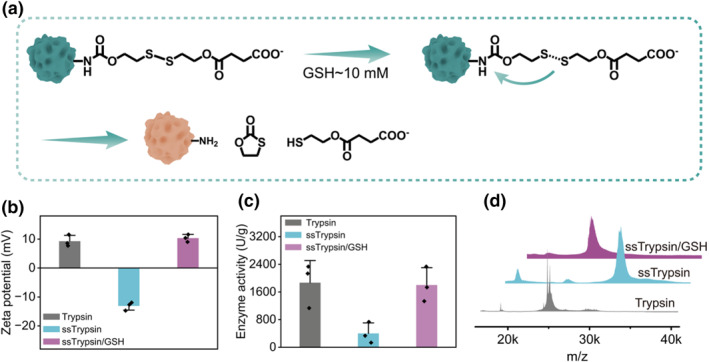
Reversible reduction response of ssTrypsin after trypsin carboxylation modification. (a) Schematic diagram of carboxylation modification of trypsin by NC‐ss‐COOH. (b) DLS analysis of the hydrodynamic size of ssTrypsin (*n* = 3). (c) Enzyme activity of ssTryspin and recovery of enzymatic activity after treatment with 10 mM GSH (*n* = 3). (d) HRMS analysis after trypsin carboxylation modification and treatment with 10 mM GSH. DLS, dynamic light scattering.

Trypsin, a representative serine protease with inherent antineoplastic activity, possesses 15 solvent‐exposed primary amines that can serve as chemoselective handles. Exploiting this feature, we conjugated NC‐ss‐COOH to the protein surface under mild aqueous conditions (0.1 M NaHCO_3_, pH 8.0) via EDC/NHS‐mediated amide coupling (Figure 1a). The resultant construct, termed ssTrypsin, was purified by size‐exclusion chromatography to remove unreacted small molecules.

Chemical transformation was verified by three complementary analytical assays. Firstly, dynamic light scattering (DLS) revealed a reversal of the zeta potential from +9.27 ± 1.65 mV (native trypsin) to −13.1 ± 1.17 mV (ssTrypsin), consistent with the introduction of multiple anionic carboxylates (Figure 1b). In addition, MALDI‐TOF MS showed a mass increment from 23,985 Da (native) to 31,042 Da (modified), corresponding to an average payload of ∼14 NC‐ss‐COOH residues per enzyme molecule (Figure 1d).

Collectively, these data establish the successful and stoichiometrically controlled carboxylation of trypsin, yielding a well‐defined prodrug ready for subsequent cisplatin coordination.

### 2.3 Redox‐triggered activation of the trypsin prodrug by cytosolic GSH

Capitalizing on the 1–10 mM cytosolic glutathione (GSH) gradient that distinguishes malignant cells from the extracellular milieu, we installed a disulfide (‐ss‐) linker within NC‐ss‐COOH to enable thiol‐responsive drug release. In the reducing intracellular environment, the disulfide undergoes rapid thiol–disulfide exchange, cleaving the macromolecular shell and restoring native enzyme activity.

Kinetics and efficiency of the redox reaction were first examined by physicochemical assays. After 12 h incubation of ssTrypsin in pH 7.4 PBS containing 10 mM GSH (37°C, 5% CO_2_), the zeta potential reverted from −13.1 ± 1.2 mV to +10.3 ± 0.2 mV (Figure 1b), mirroring the value of the unmodified protein. MALDI‐TOF MS revealed a parallel mass decrease from 31.0 kDa back to 24.1 kDa (Figure 1d), confirming quantitative release of NC‐ss‐COOH fragments. No change was observed in the absence of GSH or in 10 mM GSSG, underscoring the specificity of the disulfide cleavage.

Functional consequences of the redox event were quantified with a chromogenic trypsin activity kit (Nα‐benzoyl‐L‐arginine‐4‐nitroanilide substrate). Carboxylation appeared to silence catalytic efficiency. Exposure to 10 mM GSH restored 96% of the original activity, whereas 24 h incubation in non‐reducing buffer elicited no recovery (Figure 1c).

Collectively, these data establish that (i) NC‐ss‐COOH confers a robust, reversible silencing of trypsin, and (ii) the enzyme is re‐activated exclusively under pathophysiologically relevant reducing conditions. This proof‐of‐concept validates a generalizable strategy for constructing tumor‐microenvironment‐responsive protein therapeutics whose cytotoxic or immunomodulatory functions are spatially and temporally gated by intracellular GSH.

### 2.4 One‐step manufacture of redox‐responsive dual‐drug nanomedicine cRGD‐PEG‐PGlu‐np(sstrypsin&CDDP)

**FIGURE 2:**
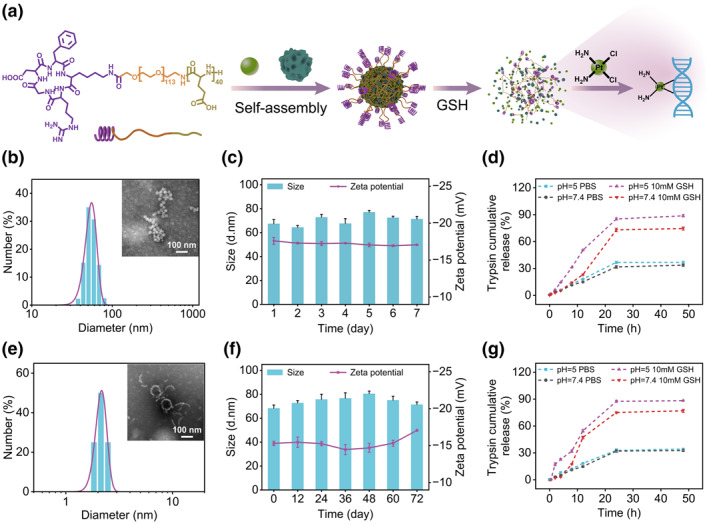
Physiochemical properties of nanomedicine of RPG‐NP (ssT&C). (a) Synchronous co‐coordination of CDDP to a carboxylated trypsin prodrug and to a multifunctional cRGD‐PEG‐PGlu block copolymer. (b) Microscopic morphologies (TEM) and hydrodynamic diameters (DLS) of RPG‐NP (ssT&C). (c) Assessment of the stability of RPG‐NP (ssT&C) in PBS buffer. (d) Release profile of CDDP with or without GSH incubation of RPG‐NP (ssT&C) under different pH milieu. (e) DLS profile and TEM image of RPG‐NP (ssT&C) incubated with 10 mM GSH. (f) The stability of RPG‐NP (ssT&C) after incubation with 10% FBS. (g) Release profile of trypsin with or without GSH incubation of RPG‐NP (ssT&C) under different pH milieu. DLS, dynamic light scattering.

A single aqueous assembly was used to entrap both cisplatin and the protease prodrug ssTrypsin within a tumor‐targeted micelle. The anionic block copolymers cRGD‐PEG‐PGlu and mPEG‐PGlu (57 Glu repeats) served as both platinum‐chelating ligands and micelle‐forming excipients. Under weakly alkaline conditions (0.1 M NaHCO_3_, pH 8.0), the γ‐carboxylates of the polyglutamate segment coordinate cisplatin in a square‐planar [Pt(OOC‐Glu)_2_Cl_2_]^2‒^ geometry, while the surface‐carboxylated trypsin is co‐inserted through complementary electrostatics and minor Pt(II) coordination (Figure 2a). A 30‐min, one‐pot process at 25°C afforded monodisperse spherical micelles: RPG‐NP (ssT&C) (cRGD‐targeted) and PG‐NP (ssT&C) (non‐targeted).

DLS gave a hydrodynamic diameter of 76.3 ± 2.2 nm (PDI = 0.12) and a zeta potential of −17.1 ± 1.2 mV for RPG‐NP(ssT&C) (Figure 2b,c). TEM revealed a regular core–corona architecture with a dry core of approximately 60 nm (Figure 2b), a size regime favorable for EPR‐mediated tumor accumulation and deep interstitial penetration.^[34]^

Drug‐loading efficiency was quantified by ICP‐MS (CDDP) and BCA assay (ssTrypsin). The micelles incorporated 11.5 ± 0.3 wt% cisplatin (loading efficiency 92%) and 3.3 ± 0.2 wt% ssTrypsin (loading efficiency 88%), corresponding to approximately 280 Pt atoms and 4 enzyme molecules per particle.

Colloidal stability was examined under both storage and physiological conditions. In PBS (4°C, 7 days), the nanoparticles retained their original size (ΔD_h_ < 5%) and zeta potential (−17 ± 1.2 mV). Incubation in 10% FBS (37°C, 72 h) produced no significant size increase (<10%) or charge reversal, indicating negligible protein corona formation (Figure 2c,f). The dense PEG corona (approximately 0.9 chains nm^−2^) and net negative surface collectively confer long‐circulating behavior.

Thus, RPG‐NP (ssT&C) integrates quantitative dual‐drug loading, tumor‐homing cRGD ligands, redox‐labile Pt(II) cross‐links and serum‐stable colloids—an optimal configuration for systemic delivery and intracellular on‐demand activation.

### 2.5 GSH‐triggered disassembly and dual‐drug release: Evidence for a redox‐synergistic mechanism

Cisplatin resistance is frequently driven by high intracellular glutathione (GSH) that scavenges the drug through Pt–SG adduct formation.^[35]^ We therefore engineered a self‐eliminating disulfide tether into the ssTrypsin surface modifier (NC‐ss‐COOH) that fulfills two complementary roles: (i) competitive consumption of cytosolic GSH, thereby protecting cisplatin from premature inactivation, and (ii) reductive cleavage that collapses the nanocarrier and synchronously releases both payloads inside tumor cells (Figure 2a).

To visualize GSH‐induced disassembly, RPG‐NP(ssT&C) was incubated overnight with 10 mM GSH. DLS disclosed a new population below 10 nm (Figure 2e), ascribed to liberated proteins and low‐molecular‐weight copolymer fragments, while TEM showed complete loss of spherical aggregates, corroborating disulfide‐mediated dissociation. Dialysis‐release studies were subsequently conducted over 48 h under sink conditions (PBS ± 10 mM GSH, pH 5.0 or 7.4, 37°C). ICP‐MS and BCA quantification revealed minimal leakage in the GSH‐free medium, attributable to slow chloride–carboxylate exchange. In contrast, 10 mM GSH provoked rapid and quantitative liberation of both drugs: at 24 h, 73% cisplatin and 75% ssTrypsin were released at pH 7.4, rising to 85% and 88%, respectively, at pH 5.0 (Figure 2d,g). The acid‐enhanced release mirrors the endo‐lysosomal microenvironment and is consistent with proton‐assisted aquation of Pt(II) complexes. Collectively, these data establish that RPG‐NP(ssT&C) undergoes GSH‐triggered disassembly and pH‐facilitated drug liberation, affording a spatiotemporally controlled co‐delivery platform for combating cisplatin‐resistant tumors.

### 2.6 Hemocompatibility and synergistic cytotoxicity: Evidence for resistance‐reversal

**FIGURE 3:**
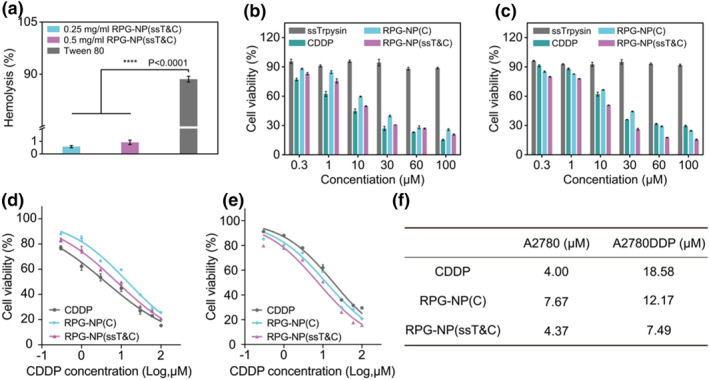
Cytotoxicity profile of RPG‐NP (ssTry&C). (a) Hemolysis percentage of nanoparticles at graded concentrations. (b) Viability of A2780DDP cells after 24 h exposure to escalating drug doses. (c) Viability of A2780 cells under identical conditions. (d–f) IC_50_ values of each formulation in A2780 and A2780DDP lines after 24 h of drug treatment. Data are mean ± SD; significance was assessed by one‐way ANOVA: *p < 0.05, **p < 0.01, ***p < 0.001, ****p < 0.0001.

Biocompatibility is a prerequisite for clinical translation. Hemolysis assay showed that RPG‐NP (ssT&C) at 0.25 and 0.5 mg mL^−1^ induced <2% RBC lysis—well below the 5% safety threshold—whereas 0.5 mg mL^−1^ Tween‐80 produced 88.5% hemolysis (Figure 3a). The negligible erythrocyte damage predicts safe intravenous administration and prolonged circulation.

We next interrogated whether co‐delivery of cisplatin and ssTrypsin could overcome platinum resistance. CCK‐8 assays were performed on parental A2780 and A2780‐DDP (resistant) cells treated with free CDDP, free ssTrypsin, single‐drug RPG‐NP(CDDP) or dual‐drug RPG‐NP(ssT&C). After 24 h of treatment, in A2780 cells, free CDDP exhibited rapid dose‐dependent toxicity (IC_50_ = 4.00 μM), whereas ssTrypsin alone was non‐cytotoxic, confirming successful enzymatic silencing. RPG‐NP(ssT&C) displayed a modest right‐shift (IC_50_ = 4.37 μM) relative to free CDDP but was significantly more potent than single‐drug nanoparticles (IC_50_ = 7.67 μM), indicating early cooperative activity (Figure 3c,d).

The benefit became dramatic in A2780‐DDP cells: free CDDP lost efficacy (IC_50_ = 18.58 μM), whereas RPG‐NP(ssT&C) retained an IC_50_ of 7.49 μM—1.6‐fold lower than single‐drug nanoparticles (12.17 μM) and 2.5‐fold lower than free CDDP (Figure 3b,e,f). The resistance index (IC_50_ resistant/IC_50_ parental) dropped from 4.6 (free CDDP) to 1.7 (RPG‐NP(ssT&C)) documenting genuine resistance reversal.

**FIGURE 4:**
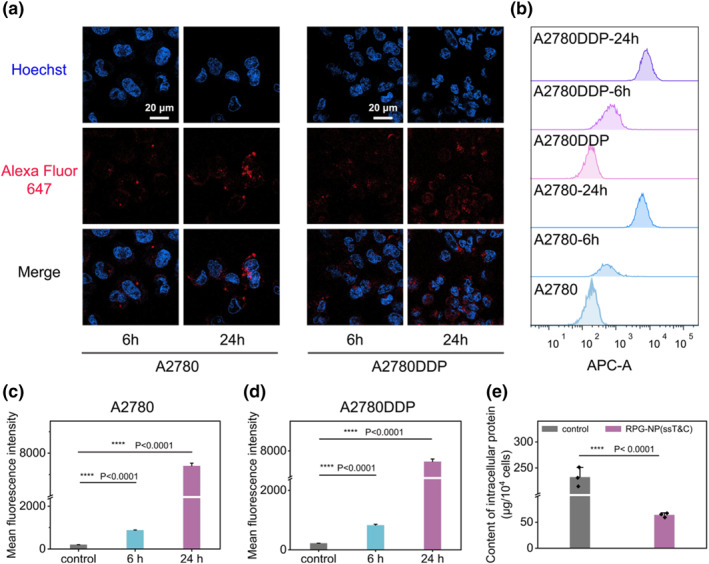
Cellular uptake and proteome depletion induced by RPG‐NP(ssTry&C). (a) CLSM images of A2780 and A2780DDP cells incubated with nanoparticles for 6 and 24 h. (b) Flow‐cytometric quantification of Alexa Fluor 647 fluorescence at the same intervals. (c, d) Mean fluorescence intensity (MFI) versus time for A2780 and A2780DDP cells, respectively. (e) Total intracellular protein content after 24 h treatment with RPG‐NP(ssTry&C). Data are mean ± SD; significance was evaluated by one‐way ANOVA: *p < 0.05, **p < 0.01, ***p < 0.001, ****p < 0.0001.

To understand the enhanced potency, we tracked nanoparticle uptake using Alexa Fluor 647‐labeled RPG‐NP (ssT&C). CLSM showed intense perinuclear fluorescence within 24 h in both A2780 and A2780‐DDP cells (Figure 4a). Flow‐cytometry quantification revealed a 3.2‐fold increase in mean fluorescence intensity between 6 and 24 h (Figure 4b–d), indicating efficient, time‐dependent internalization that circumvents the reduced influx characteristic of resistant cells. Collectively, the data demonstrate that the nanomedicine combines safe blood contact, glutathione‐triggered drug liberation, and synergistic Pt/protease toxicity to surmount cisplatin resistance.

Furthermore, to link the observed cytotoxic synergy to a molecular mechanism, we quantified the global intracellular proteome 24 h after treatment. BCA assay showed that RPG‐NP(ssT&C) reduced total protein content in A2780 cells by approximately 73% versus untreated controls (Figure 4e). This collapse mirrors the canonical activity of trypsin and demonstrates that the glutathione‐activated protease efficiently degrades cytoskeletal, metabolic and DNA‐repair proteins that underpin tumor‐cell survival and platinum resistance. The proteolytic erosion of the intracellular protein network therefore provides a direct biochemical rationale for the 2.5‐fold potency gain observed in resistant cells and highlights the unique advantage of combining a DNA cross‐linker with a network‐dismantling protease.

### 2.7 Prolonged circulation and cRGD‐directed tumor accumulation of RPG‐NP(ssT&C)

**FIGURE 5:**
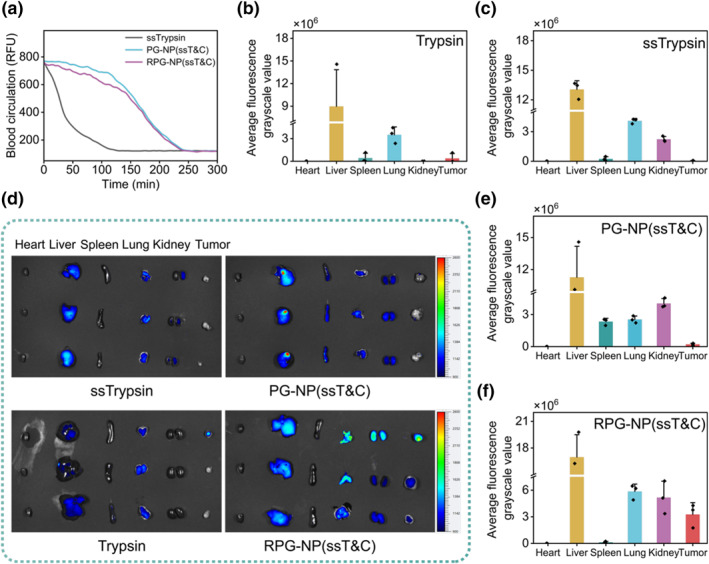
In vivo pharmacokinetics and biodistribution of RPG‐NP (ssT&C). (a) Real‐time circulation profiles in rats following i.v. administration of Alexa Fluor 647‐labeled ssTrypsin, PG‐NP (ssT&C) and RPG‐NP(ssT&C); fluorescence intensity is expressed as% of *t* = 0. (b–f) Ex vivo fluorescence images and corresponding quantification of major organs plus tumors 24 h post‐injection: (b) free trypsin, (c) free ssTrypsin, (e) PG‐NP (ssT&C), and (f) RPG‐NP(ssT&C) (*n* = 3). (d) Representative whole‐organ fluorescence image montage for all formulations at 24 h.

Protein therapeutics are rapidly cleared by renal filtration and proteolysis; therefore, extending circulation half‐life is essential for efficacy. Alexa Fluor 647‐labeled ssTrypsin, PG‐NP(ssT&C) and RPG‐NP(ssT&C) were administered intravenously to healthy female rats and tracked in real time with a fiber‐optic confocal imaging system. Free ssTrypsin disappeared exponentially (*t*_1/2_ = 35 min; 62% loss within 50 min), whereas both nanoparticle formulations exhibited markedly retarded clearance (*t*_1⁄2_ ≈ 175–177 min, Figure 5a). The five‐fold extension in circulation time provides a pharmacokinetic window compatible with tumor targeting and EPR‐mediated extravasation.

To quantify site‐specific delivery, A2780‐tumor‐bearing mice received a single tail‐vein dose of the same fluorescent conjugates. After 24 h, organs and tumors were excised and imaged ex vivo. All formulations showed prominent hepatic signals—an expected consequence of reticuloendothelial clearance—but only RPG‐NP(ssT&C) produced intense fluorescence within the tumor (Figure 5b–f). Quantitative region‐of‐interest analysis revealed an 116‐fold higher tumor exposure for RPG‐NP(ssT&C) versus free ssTrypsin (*p* < 0.001), and a 15‐fold advantage over the non‐targeted PG‐NP(ssT&C), confirming that cRGD‐mediated integrin binding superimposes active targeting onto the passive EPR effect.^[36]^ Collectively, the prolonged systemic residence and ligand‐directed intratumoral accumulation furnish a robust pharmacological basis for the superior antitumor activity observed in subsequent efficacy studies.

### 2.8 In vivo anti‐tumor therapy and immune‐microenvironment remodeling

**FIGURE 6:**
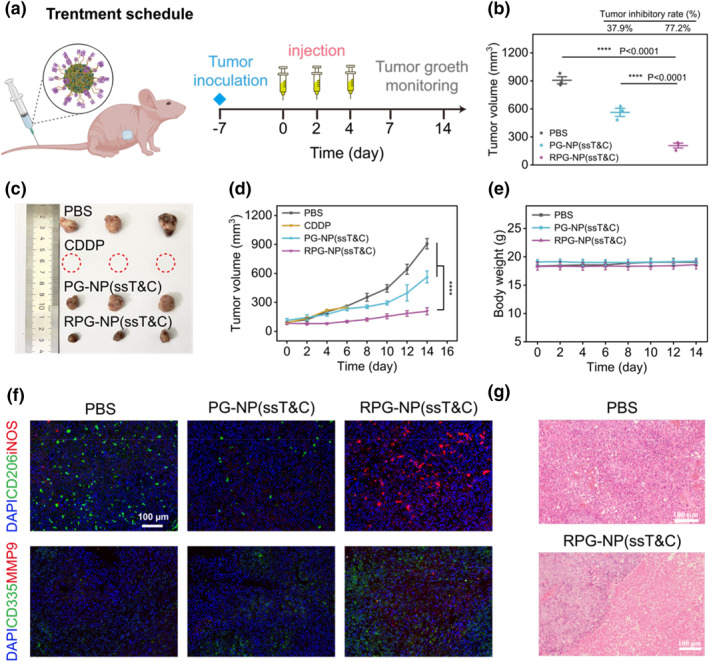
In vivo anti‐tumor efficacy and immune‐microenvironment remodeling after intravenous administration. (a) Timeline for A2780 xenograft establishment and every‐other‐day tail‐vein dosing. (b) Tumor‐inhibition rate at endpoint (*n* = 3). (c) Photograph of excised tumors on day 14. (d) Tumor‐volume kinetics during treatment (*n* = 3). (e) Body‐mass evolution over 14 days (*n* = 3). (f) Immunofluorescence micrographs of tumor sections showing iNOS^+^ M1 macrophages, MMP9 expression and CD335^+^ NK‐cell infiltration; scale bar = 50 μm. (g) H&E‐stained tumor sections illustrating necrotic/apoptotic areas; scale bar = 100 μm. Data are mean ± SD; significance was determined by one‐way ANOVA: *p < 0.05, **p < 0.01, ***p < 0.001, ****p < 0.0001.

Encouraged by the prolonged circulation and ligand‐directed accumulation of RPG‐NP(ssT&C), we evaluated therapeutic efficacy in sub‐cutaneous A2780 ovarian‐cancer‐bearing BALB/c nude mice (initial volume ≈ 100 mm^3^). Animals received i.v. injections every 2 days (PBS, free CDDP, PG‐NP(ssT&C) or RPG‐NP(ssT&C); *n* = 3) for a total of three doses (Figure 6a). After 14 days, RPG‐NP(ssT&C) produced a 77% reduction in mean tumor volume versus PBS—2.0‐fold superior to the non‐targeted PG‐NP(ssT&C) (38% inhibition) and accompanied by extensive necrosis, nuclear shrinkage and chromatin fragmentation on H&E (Figure 6b–g). In contrast, all mice given free CDDP succumbed by day 6, whereas nanoparticle‐treated groups showed no weight loss or histological lesions in heart, liver, spleen, lung or kidney (Figure 6e, Figure S13), underscoring the safety advantage of carrier‐mediated delivery.

Immunofluorescence profiling of excised tumors revealed that RPG‐NP(ssT&C) profoundly remodeled the immune microenvironment. Compared with PBS controls, treated tumors displayed dense infiltration of iNOS^+^ M1 macrophages and elevated MMP9 expression (Figure 6f), consistent with pro‐inflammatory polarization and extracellular‐matrix degradation. Concomitantly, CD335^+^ natural killer cells were markedly enriched,[^37^, ^38^] suggesting activation of innate cytotoxic pathways (perforin/granzyme, IFN‐γ). Thus, the nanomedicine not only delivers cisplatin and protease in a synergistic ratio but also converts an immunologically “cold” tumor into an inflamed, drug‐sensitive niche, providing a mechanistic rationale for its superior anti‐tumor activity.

## 3 CONCLUSIONS

In summary, this study established a dual‐drug co‐delivery platform that combines targeting, stability, and intelligent drug release functions, maintaining the safety of normal tissues while generating synergistic antitumor effects through orthogonal mechanisms of action. Using cRGD tumor‐targeting peptide‐modified polyethylene glycol ‐polyglutamic acid (cRGD‐PEG‐PGlu) as the carrier, we successfully integrated CDDP and surface carboxylated trypsin (ssTrypsin) to construct dual‐drug nanoparticles (RPG‐NP (ssT&C)) via electrostatic interactions and co‐coordination self‐assembly techniques. These nanoparticles exhibit a uniform spherical structure (approximately 76.3 nm) and significantly enhance drug accumulation at tumor sites through integrin receptor‐mediated active targeting mechanisms. Following endocytosis, the nanoparticles precisely release drugs in the high glutathione (GSH) microenvironment of tumors while effectively depleting GSH to inhibit the formation of Pt‐GSH complexes. In vivo anti‐tumor evaluations demonstrated that after three tail vein administrations, RPG‐NP (ssT&C) exhibited a significant tumor inhibitory effect (inhibition rate of 77.2%) in the A2780 ovarian cancer model, with toxicity assessments confirming no significant damage to major organs. In summary, the intelligent nanodelivery system developed in this study effectively overcomes the challenge of chemotherapy resistance in ovarian cancer through multi‐mechanism synergistic action, significantly enhancing anti‐tumor efficacy while ensuring treatment safety. This provides a new strategy with important translational value for the clinical treatment of ovarian cancer, showcasing broad clinical application prospects.

## CONFLICT OF INTEREST STATEMENT

The authors declare no conflicts of interest.

## ETHICS STATEMENT

All protocols for animal studies conformed to the Guide for the Care and Use of Laboratory Animals and approved by the Dalian University of Technology Animal Care and Use Committee (DUT2020‐028).

## Supporting information

Supporting Information S1

## Data Availability

The data that supports the findings of this study are available in the supplementary material of this article.
