# Supplementary material for: Redox‐responsive dual‐drug nanomedicine integrating cisplatin and trypsin for synergistic reversal of tumor chemoresistance
Source: Smart Mol. 2026 Apr 23:e70055. Online ahead of print. doi: 10.1002/smo2.70055 (PMC13398630; doi:10.1002/smo2.70055)
Supplement: Supplementary file 1 — Supporting Information S1 [file SMO2-9999-0-s001.docx]

**Supporting Information**

**Redox-responsive dual-drug nanomedicine integrating cisplatin and trypsin for synergistic reversal of tumor chemoresistance**

Xiaolan Yin ^a, b, #^, Qi Wang ^a, b, #^, Ming Zhang ^a^, Cheng Zhang ^a^, Qixian Chen^c,*^, Haidong Li ^a^, Yan Zhao^d,e,f^, Yue Wang^d,e,f^, Jingyun Wang ^a,*^, Liuwei Zhang ^b,*^, Hongyan Cui ^b,*^

^#^These authors contributed equally to this work.

^a^School of Bioengineering, Dalian University of Technology, 2 Linggong Road, Dalian 116024, China. E-mail: wangjingyun67@dlut.edu.cn (J. Wang).

^b^Innovation Center of Yangtze River Delta, Zhejiang University, Jiaxing 314100, China. E-mail: cuihongyan@zju.edu.cn (H. Cui), zhanlw@zju.edu.cn (L. Zhang)

^c^Provincial Key Laboratory of Interdisciplinary Medical Engineering for Gastrointestinal Carcinoma, Liaoning Cancer Hospital & Institute, No. 44 Xiaoheyan Road, Dadong District, Shenyang 110042, China. E-mail: plasmid@zju.edu.cn (Q. Chen).

^d^Department of Gastric Surgery, Cancer Hospital of Dalian University of Technology, No. 44 Xiaoheyan Road, Dadong District, Shenyang 110042, China

^e^Department of Gastric Surgery, Cancer Hospital of China Medical University, No. 44 Xiaoheyan Road, Dadong District, Shenyang 110042, China

^f^Provincial Key Laboratory of Interdisciplinary Medical Engineering for Gastrointestinal Carcinoma, Liaoning Cancer Hospital & Institute, No. 44 Xiaoheyan Road, Dadong District, Shenyang 110042, China

***1. Materials***

α-Methoxy-ω-amino-polyethylene glycol (mPEG-NH_2_, *M*_w_ 5000 Da) and tert-Butoxycarbonyl-iminopolyethylene glycol-activated ester (BOC-NH-PEG-NHS, M_w_ 5000 Da) were purchased from SinoPEG Co. (Xiamen, China). Glutamic acid 5-benzyl ester N-carboxycyclic endoic anhydride (L-Glu(Obzl)-NCA) was purchased from Sichuan Jiayinglai Technology Co., Ltd. Tumor-targeting peptide cRGDfk was purchased from Nanjing Yuanpeptide Biotechnology Co., Ltd. Alexa Fluor 647 NHS ester (succinimidyl ester) was purchased from Thermo Fisher Scientific, Inc. (China). Matrix gel and Cell Counting Kit-8 (CCK8) were purchased from Yeasen Biotechnology (Shanghai) Co., Ltd. RIPA lysate was purchased from Beyotime Company (China). 4-Nitrophenyl chloroformate, 1,6-Hexanediol, reduced GSH and Cisplatin (CDDP) were purchased from Aladdin Biochemical Technology Co., The nucleic dye Hoechst 33342 and BCA Protein Quantification Kit were purchased from Beijing Suolarbao Technology Co., Ltd. Trypsin was purchased from Sigma-Aldrich (USA). Anti-iNOS, Anti-NCR1, Anti-CD206, Anti-MMP9 were purchased from Sevier Biotechnology Co. (Wuhan, China), Paraformaldehyde was purchased from Shanghai Titan Technology Co. All other chemical reagents used in this work were analytically pure.

***2. Cell Lines and cell Culture***

Human ovarian cancer cells A2780 and its cisplatin-resistant strain A2780DDP were cultured in vitro using RPMI-1640 medium (containing 10% fetal bovine serum, 1% double antibody: penicillin 100 U/mL, streptomycin 100 µg/mL).

***3. Animal experiments***

Female 4-5 weeks old Bal b/c nude mice (16-18 g) were obtained from Beijing Vital River Laboratory Animal Technology Co., Ltd. The mouse tumor model was established by subcutaneous injection of A2780 cells, and the tumor growth process was continuously monitored. The management and operation procedures of experimental animals strictly follow the ethical standards of the "Guidelines of the Care and Use of Laboratory Animals formulated" published by the Ministry of Science and Technology of China, and have been approved by the Experimental Animal Ethics Committee of Dalian University of Technology (DUT2020-028).

***4. Synthesis of NC-ss-OH***

NC-ss-OH was synthesized by acylation reaction between 1,6-hexanediol and p-nitrophenyl chloroformate. To prevent the oxidation and hydrolysis of the reaction intermediates, the reaction was conducted under anhydrous, anaerobic, and inert conditions. Inside an argon-filled glove box, 1,6-hexanediol (0.8 g, 5.2 mmol) was dissolved in a dichloromethane solution (DCM). After complete dissolution, p-nitrophenyl chloroformate (0.524 g, 2.6 mmol) was added. Once the reactants were thoroughly mixed, anhydrous triethylamine (0.707g, 7 mmol) was slowed added under a nitrogen purge to enhance the efficiency of the nucleophilic substitution reaction. Finally, the reaction mixture was stirred at room temperature overnight (Scheme S1).

After the reaction was confirmed to be complete using thin layer chromatography (TLC), the resulting suspension was transferred to a rotary evaporator to remove the solvent DCM, yielding a yellow oily crude product. Subsequently, the crude product was dissolved in ethyl acetate under low temperature conditions, and the precipitated byproduct, triethylamine hydrochloride, was filtered out. Finally, the resulting mixture was purified using silica gel chromatography, employing a gradient elution with a mixture of dichloromethane and ethyl acetate (20:1 v/v), and the product NC-ss-OH was obtained after purification.

***5. Synthesis of NC-ss-COOH***

NC-ss-COOH was synthesized by esterification reaction between NC-ss-OH and succinic anhydride. Similarly, the reaction was conducted under a nitrogen purge. Initially, NC-ss-OH (400 mg, 1.25 mmol) was dissolved in anhydrous tetrahydrofuran (THF). Anhydrous triethylamine (80 mg, 0.80 mmol) was then added dropwise, and after thorough mixing, succinic anhydride (250 mg, 2.5 mmol) was introduced. The reaction vessel was sealed and stirred at room temperature for 24 hours (Scheme S2).

. After the reaction was confirmed to be complete using TLC, the resulting suspension was transferred to a rotary evaporator to remove the solvent THF, yielding a yellow oily crude product. Subsequently, the crude product was dissolved in DCM and washed multiple times with saturated saline solution to remove unreacted succinic anhydride and triethylamine. The organic phase was collected and dried over anhydrous sodium sulfate. Finally, the crude product was purified using silica gel column chromatography, employing a mixture of DCM and anhydrous methanol in a volume ratio of 40:1 as the eluent, resulting in the purified product NC-ss-COOH.

***6. Synthesis of the polyanionic cRGD-PEG-PGlu***

The synthesis of the polyanionic block copolymer cRGD-PEG-PGlu was conducted through two steps (Scheme S3). The first step was the conjugation of the amino group of cRGD peptide with BOC-NH-PEG-NHS. Initially, RGD (54.3 mg, 90 µmol) was added to a NaHCO_3_ buffer solution (pH 8.4, 0.1M) and stirred thoroughly to activate the RGD peptide. Subsequently, BOC-NH-PEG-NHS (200 mg, 37.8 µmol) was added under ice bath conditions, and the reaction was allowed to proceed at room temperature for 12 hours. The resulting mixture was then dialyzed three times against ultrapure water using a dialysis bag with a molecular weight cutoff of 5 kDa to remove unreacted starting materials and by-products, yielding aqueous solution of cRGD-PEG-NH-BOC. In order to expose the amino group of cRGD-PEG-NH-BOC for the next reaction step, dilute hydrochloric acid was added to the aqueous solution of cRGD-PEG-NH-BOC to remove the BOC protecting group. After a 12-hour reaction at room temperature, the solution was dialyzed again using a 5 kDa molecular weight cutoff dialysis bag to eliminate the acid and by-products. Following dialysis, the product was freeze-dried to obtain cRGD-PEG-NH_2_. The conjugation efficiency of cRGD with PEG was determined by analyzing the characteristic peak signals in the ^1^H-NMR spectrum (Figures S7, S8).

The second step in the synthesis of cRGD-PEG-PGlu was achieved by ring-opening polymerization of cRGD-PEG-NH_2_ with L-Glu (Obzl)-NCA monomer. Initially, cRGD-PEG-NH2 (100 mg, 17.6 µmol) was dissolved in DCM and an excess of benzene was added, and freeze-dried for water removal. Subsequently, cRGD-PEG-NH_2_ was dissolved in DMF in a glove box under argon atmosphere with stirring to fully dissolve, L-Glu (Obzl)-NCA (290 mg, 1.10 mmol) was added, and the reaction was carried out at 30 °C for 72 hours. After the reaction was completed, unreacted raw materials and by-products were removed by washing several times with ice ether, and the lower precipitate was retained to remove residual ether using vacuum drying. The resulting product cRGD-PEG-PGlu (Obzl) was dissolved in a methanol solution of 1 M NaOH and reacted at room temperature for 24 hours to remove the protective groups. Subsequently, the reaction solution was transferred to a dialysis bag with a molecular weight cut-off of 10 K and dialyzed three times in 0.01 M hydrochloric acid and ultrapure water, and lyophilized to obtain the product cRGD-PEG-PGlu (Scheme S4).

***7. Synthesis of the polyanionic mPEG-PGlu***

Polyanionic carrier PEG-PGlu without cRGD targeting peptide was synthesized according to the synthesis method of cRGD-PEG-PGlu. First, mPEG-NH_2_ (100 mg, 20 µmol) was dissolved in DCM and an excess of benzene was added, and freeze-dried for water removal. Subsequently, in an argon-filled glove box, mPEG-NH_2_ was dissolved in DMF and stirred until fully dissolved, L-Glu (Obzl)-NCA (290 mg, 1.10 mmol) was added, and the reaction was carried out at 30 °C for 72 hours. After the reaction was completed, unreacted raw materials and by-products were removed by washing several times with ice ether, and the lower precipitate was retained to remove residual ether using vacuum drying. The resulting product mPEG-PGlu (Obzl) was dissolved in a methanol solution of 1 M NaOH and reacted at room temperature for 24 h to remove the protective groups. Subsequently, the reaction solution was transferred to a dialysis bag with a molecular weight cut-off of 10 K and dialyzed three times in 0.01 M hydrochloric acid and ultrapure water, and lyophilized to obtain the product mPEG-PGlu (Scheme S5).

***8. Carboxylation modification of proteins via NC-ss-COOH***

The carboxylation modification of protein (trypsin as an example) was achieved by using the amino group present on the protein surface via the formation of an amide bond between the amino group and the terminal carboxyl group of NC-ss-COOH under weakly alkaline conditions. Trypsin (10 mg, 0.42 µmol) was dissolved in NaHCO_3_ buffer (10 mL, 0.1 M) and stirred continuously at 4℃ for 30 minutes to ensure complete dissolution. Subsequently, NC-ss-COOH, at a quantity equivalent to 100 equivalents of protein amino groups, was dissolved in a small amount of DMSO and slowly added dropwise to the protein buffer solution. The reaction was maintained at 4℃ with continuous stirring overnight. After the reaction was complete, unreacted small molecules were selectively removed using a centrifugal ultrafiltration device with a molecular weight cutoff of 3 kDa, through multiple cycles of centrifugation (4000 g, 4 °C, 15 min/time), and the cutoff solution was collected to obtain the purified product ssTrypsin. The concentration of ssTrypsin was then measured using a BCA protein assay kit and adjusted to a concentration of 1 mg/ml. The degree of carboxylation modification of the protein was determined using matrix-assisted laser desorption/ionization time-of-flight mass spectrometry and dynamic light scattering (DLS) analyzer.

***9. Preparation of nanoparticles RGD-PEG-PGlu-NP (ssTrypsin&CDDP), PEG-PGlu-NP (ssTrypsin&CDDP) and RGD-PEG-PGlu-NP(CDDP)***

Cisplatin forms hydrated cisplatin in aqueous solution, which coordinates with carboxyl groups of twice the molecular weight to create drug-loaded nanoparticles. The synthesized cRGD-PEG-PGlu (10.8 mg, 1.0 µmol) and CDDP (12 mg, 40 µmol) were firstly co-dissolved in 10 mL of deionized water and stirred thoroughly to make the mixture homogeneous. Subsequently, ssTrypsin (4 mg, 0.17 µmol) was added and the pH was adjusted to 8.0. The reaction system was continuously stirred at 37 ℃ for 72 h to ensure adequate coordination of hydrated cisplatin to the carboxyl group of cRGD-PEG-PGlu and to achieve effective loading of trypsin. After the reaction was completed, the reaction solution was dialyzed using a dialysis bag with a molecular weight cut-off of 100 K to remove unbound CDDP and ssTrypsin, and finally the aqueous dispersion of RGD-PEG-PGlu-NP (ssTrypsin&CDDP) (referred to as RPG-NP (ssT&C)) was obtained.

As a control, two types of nanomedicine were prepared using the same methods and material ratios: RGD-PEG-PGlu-NP(CDDP) (referred to as RPG-NP (CDDP)), which is a tumor-targeting nanoparticle containing only CDDP and does not encapsulate ssTrypsin; and PEG-PGlu-NP (ssTrypsin&CDDP) (referred to as PG-NP (ssT&C)), which does not contain the RGD targeting peptide but is loaded with CDDP and ssTrypsin. Ultimately, all nanoparticles were concentrated using ultrafiltration tubes (MWCO: 3 kDa) to enhance micelle concentration and remove residual small molecules.

***10. Physicochemical characterizations of RPG-NP (ssT&C)***

The physicochemical properties of RPG-NP (ssT&C) (1 mg/ml) were systematically evaluated at 25 °C in 10 mM PBS buffer. The hydrodynamic size distribution of RPG-NP (ssT&C) was evaluated using dynamic light scattering (DLS) measurement with a Zetasizer Nano 90. In order to evaluate its stability, the particle size distribution and zeta potential were measured over a period of 7 days in PBS buffer. Additionally, RPG-NP (ssT&C) was incubated in 10% FBS, and the particle size distribution and zeta potential were monitored over 72 hours to assess its serum stability. The morphology of RPG-NP (ssT&C) was observed using transmission electron microscopy (TEM) with a Tecnai G2 Spirit operating at an acceleration voltage of 125 kV, after staining with phosphotungstic acid as a negative stain.

***11. Redox response and drug release of RPG-NP (ssT&C)***

To investigate the responsiveness of the dual-drug nanoparticles RPG-NP (ssT&C) to a reducing environment, RPG-NP (ssT&C) was incubated overnight at 37°C with 10 mM GSH. The morphological changes of the nanoparticles were observed using TEM, and the particle size and zeta potential of RPG-NP (ssT&C) were measured using DLS.

Furthermore, to evaluate the redox-responsive drug release of RPG-NP (ssT&C), the release amounts of Trypsin and CDDP were determined using a dialysis method under specific pH conditions and varying concentrations of GSH. Four buffer systems were established to simulate specific physiological scenarios: pH 5 with or without 10 mM GSH, and pH 7 with or without 10 mM GSH. Subsequently, 5 mL of RPG-NP (ssT&C) (1 mg/mL) was added to the dialysis bag with a molecular weight cutoff of 100 kDa and immersed in the corresponding buffer solution (60 mL) while stirring at 37 °C. At various time points over 48 hours, 1 mL of the release medium was collected, and an equal volume of fresh buffer was added to maintain a constant volume. The released amounts of Trypsin and CDDP were quantified using the BCA method and ICP-MS, respectively.

***12. Hemolysis assay for RPG-NP(ssT&C)***

Hemocompatibility of RPG-NP(ssT&C) was assessed by determining the hemolysis rate of erythrocytes by sterile defibrinated sheep blood. Sterile sheep blood was centrifuged at 1000 g under 4°C to collect the precipitated red blood cells, which were then resuspended in PBS (pH 7.4, 10 mM). This washing process was repeated until the supernatant was clear, after which the red blood cells were resuspended in PBS to achieve a final concentration of 2% (v/v). Subsequently, the erythrocyte suspension was mixed with different concentrations of RPG-NP (ssT&C) (0.25 and 0.5 mg/ml) in equal volumes. Tween 80 was used as a positive control. The mixtures were incubated at 37℃ for 4 hours, followed by followed by centrifugation to collect the supernatant. The absorbance at 540 nm was measured using a microplate reader to evaluate the potential hemolytic effect of the modified RPG-NP (ssT&C) under physiological conditions. The hemolytic rate was calculated using the following formula:

Hemolytic rate (%) = (OD sample – OD negative control) / (OD positive control - OD negative control) × 100%.

***13. Evaluation of cytotoxicity and drug resistance***

The in vitro cytotoxicity of RPG-NP (ssT&C) on A2780 and A2780DDP cells was evaluated using Cell Counting Kit-8 reagent. A2780 cells and A2780DDP cells were seeded in 96-well plates at a density of 1×10^5^ cells/mL and incubated in a cell culture incubator at 37°C with 5% CO_2_ for 24 h to promote cell proliferation. The initial culture medium was discarded and replaced with fresh medium containing different concentrations of CDDP, ssTrypsin, RPG-NP (CDDP), and RPG-NP (ssT&C) for continued incubation for 24 hours.

Subsequently, the drug medium was replaced with a medium containing 10% concentration of CCK8 and the incubation was continued for 4 h. During this process, enzymes in the living cells reduced WST8 to the highly water-soluble yellow formazan. The optical density (OD) value was measured at 450 nm using a microplate reader. The untreated group served as the negative control, and each experimental group consisted of five replicates. Cell viability was calculated using the following formula:

Cell viability (%) = [(OD sample – OD blank control)/ (OD control – OD blank control)] × 100%.

The evaluation of cellular drug resistance was determined by inferring the critical IC50 value from the CCK8 cytotoxicity curve of cisplatin-resistant A2780DDP cells, indicating the concentration of drug required to inhibit the cells growth by 50%. The improvement effect of nanoparticles on drug resistance was verified by calculating the IC_50_. The drug resistance reversal index of the cells was calculated according to the following equation:

Drug Resistance Reversal Index = IC₅₀ value of A2780DDP (free drug group) / IC₅₀ value of A2780DDP (nanoparticle group)

***14. Cellular uptake of RPG-NP (ssT&C)***

The uptake efficiency and distribution of dual-drug nanoparticles in A2780 and A2780DDP cells were comprehensively evaluated using confocal laser scanning microscopy (CLSM) and flow cytometry (FCM). In this study, RPG-NP (ssT&C) was conjugated with Alexa Fluor 647 NHS ester for drug tracing. A2780 and A2780DDP cells were inoculated in confocal dishes at a density of 1 × 10⁴ cells/mL and cultured in adherence at 37°C and 5% CO₂ for 24 h. The original medium was replaced with fresh medium containing Alexa Fluor 647 NHS-labeled nanoparticles, and the cells were further cultured for 6 h or 24 h to assess cell endocytosis and distribution. At the end of culture, the cells were stained with 1 µL of Hoechst 33342 nuclear dye to facilitate observation of cellular structure, washed with PBS and analyzed by (CLSM) for multichannel imaging.

To quantify the endocytosis of nanoparticles, A2780 and A2780DDP cells were seeded at the same density as described above and incubated in 6-well plates for 24 h to allow for cell proliferation. Subsequently, drugs were added, and the cells were co-cultured for either 6 hours or 24 hours. Following this, the cells were digested with trypsin and collected, thoroughly washed with PBS, and then resuspended in 400 µL of PBS. FCM was employed for quantitative analysis to determine the drug uptake efficiency.

***15. In vivo pharmacokinetics and biodistribution***

To illustrate the pharmacokinetic behavior of nanodrugs in vivo and provide deeper insights into the pharmacokinetics of dual-drug nanoparticles, Alexa Fluor 647 NHS-labeled drugs were administered via tail vein injection into healthy female rats (injection dose: 1 mg/kg). The metabolic dynamics of the drug in the rat bloodstream were observed in real-time using fiber-optic in vivo confocal microscopy to obtain time-dependent pharmacokinetic distribution curves.

To further investigate the distribution of nano-drugs in animals post-administration, A2780 cells were cultured and subcutaneously inoculated at a density of 5×10^6^ cells per mouse into the axillae of female BALB/c nude mice. Once the tumor volume reached approximately 150 mm^3^, the mice were randomly divided into four groups: Trypsin, ssTrypsin, PG-NP (ssT&C), and RPG-NP (ssT&C). The mice were euthanized 24 hours after administration via tail vein injection (with CDDP at a dosage of 3 mg/kg and Trypsin at 1 mg/kg), and tumors along with major organs (heart, liver, spleen, lungs, and kidneys) were harvested. The distribution and the fluorescence intensity of the drug at each tissue were observed and quantified using an in vivo imaging system to assess the distribution.

***16. In vivo anti-tumor therapy***

The in vivo antitumor effects of drugs were evaluated by establishing an ovarian cancer model subcutaneously in mice. A2780 cells were cultured and adjusted to a density of 5×10⁶, mixed with matrix gel and subcutaneously inoculated into the right axilla of female Bal b/c nude mice. Once the tumor volume reached approximately 100 mm³, the mice were randomly divided into four groups and treated via tail vein injection. Throughout the treatment period, the tumor volume and body weight of the mice were monitored regularly. Tumor volume (V) was calculated using the formula V = a × b² × 0.5, where a and b represent the length and width of the tumor, respectively. The tumor volume suppression rate is calculated using the formula [(V_PBS_ – V_treatment_) / V_PBS_] × 100%, comparing the treatment group with the PBS control group at the same time point to evaluate antitumor efficacy. After 14 days of treatment, the mice were euthanized, and tumor tissues along with major organs (hearts, livers, spleens, lungs, kidney) were collected for histopathological assessment using hematoxylin and eosin (H&E) staining.

In addition, immunofluorescence staining was performed to analyze the infiltration of tumor-associated natural killer (NK) cells and tumor-associated macrophages (TAMs) in the tumor tissue. The degradation status of the tumor microenvironment was also assessed to evaluate the proteolytic effects of proteases on the extracellular matrix (ECM), thereby determining the potential immunotherapeutic efficacy of the drug treatment.

**Scheme S1.** Synthesis routes of NC-ss-OH.

**Scheme S2.** Synthesis route of NC-ss-COOH.

**Scheme S3.** Synthesis route of RGD-PEG-NH_2_.

__

**Scheme S4.** Synthesis routes of RGD-PEG-PGlu.

**Scheme S5.** Synthesis routes of PEG-PGlu.

**Supplementary Figures**


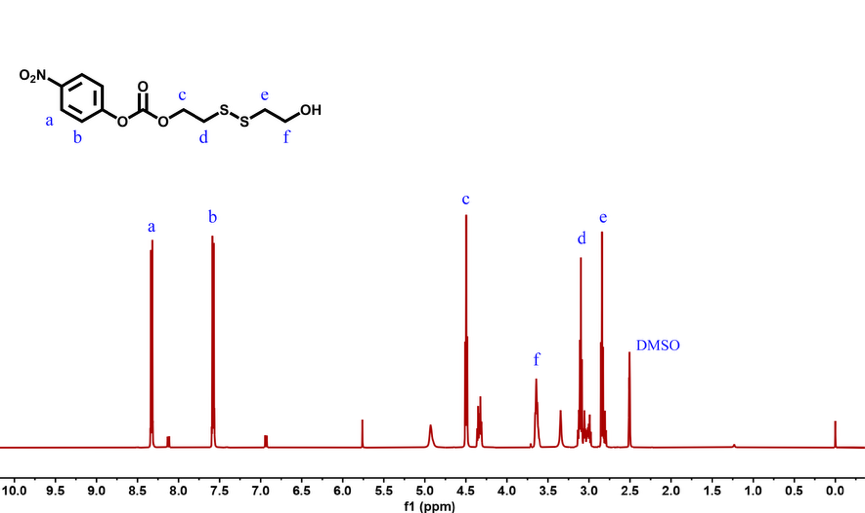


**Figure S1.** ^1^H-NMR spectrum of NC-ss-OH in DMSO-d6.


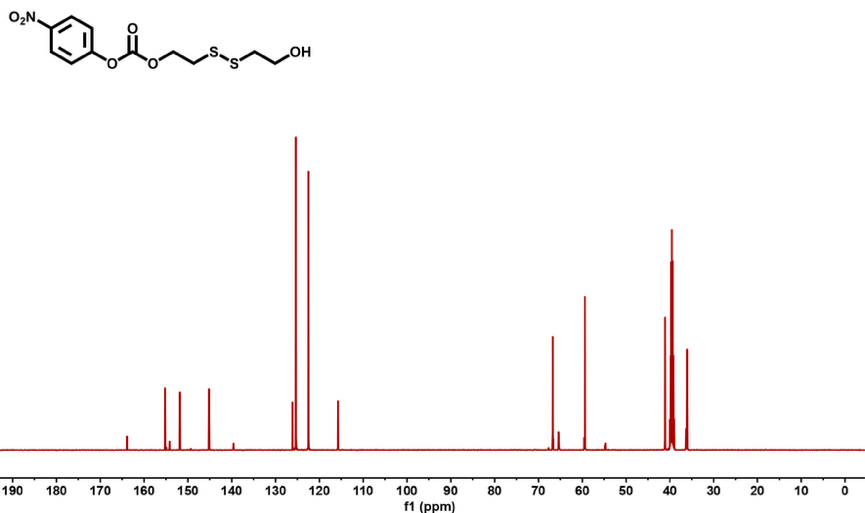


**Figure S2.** ^13^C-NMR spectrum of NPC-ss-OH in DMSO-d6.


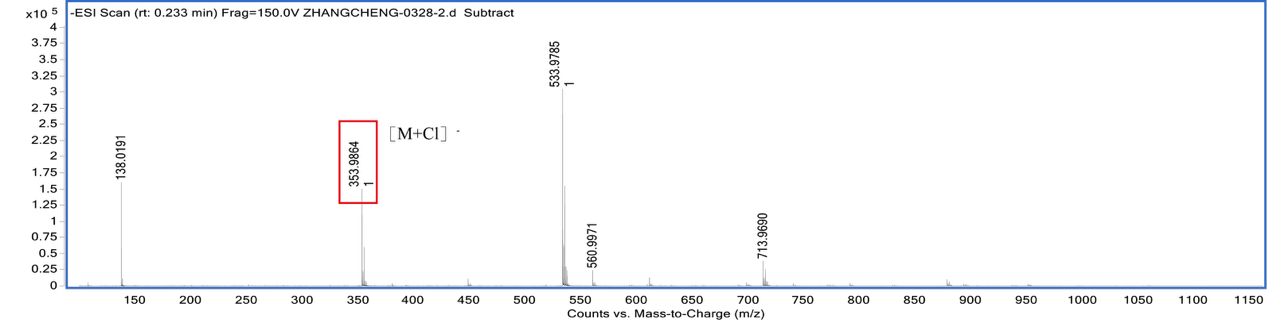


**Figure S3.** HRMS spectrum of NC-ss-OH.


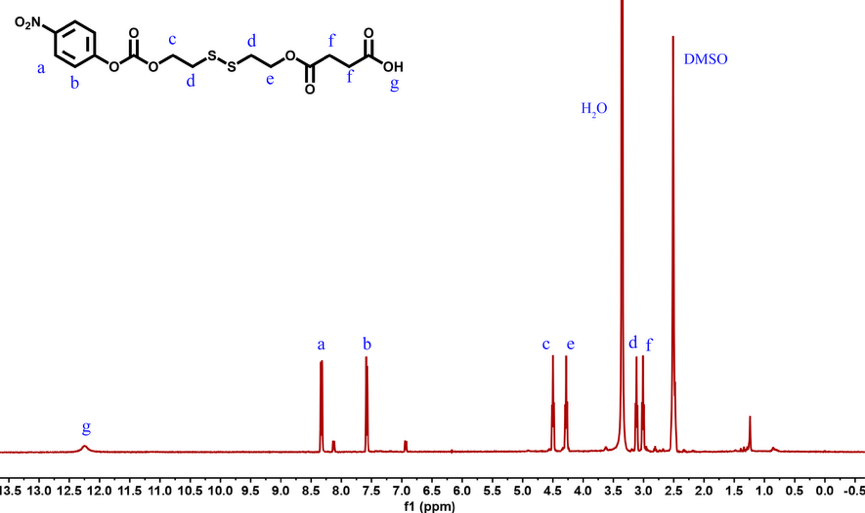


**Figure S4.** ^1^H-NMR spectrum of NC-ss-COOH in DMSO-d6.


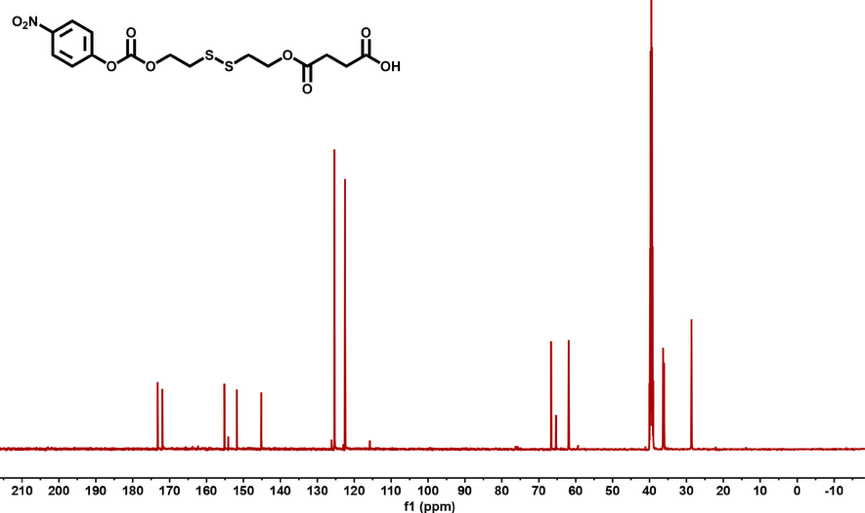


**Figure S5.** ^13^C-NMR spectrum of NC-ss-COOH in DMSO-d6.


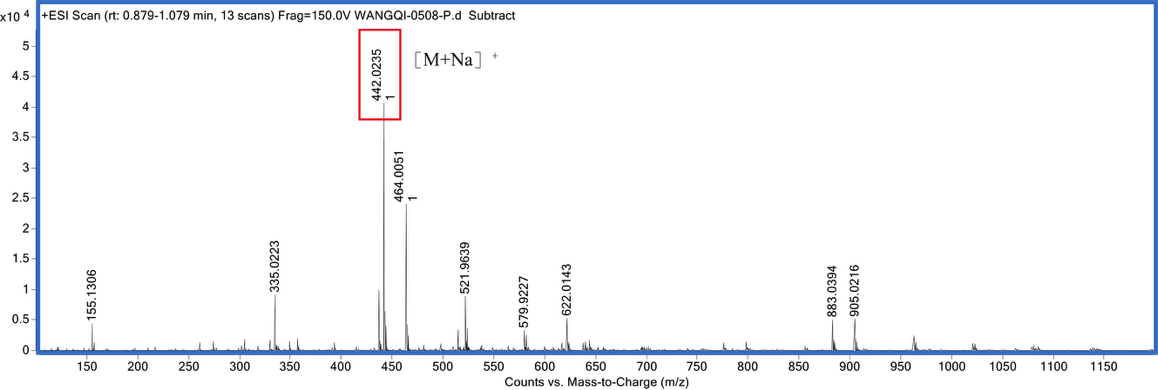


**Figure S6.** HRMS spectrum of NC-ss-COOH.


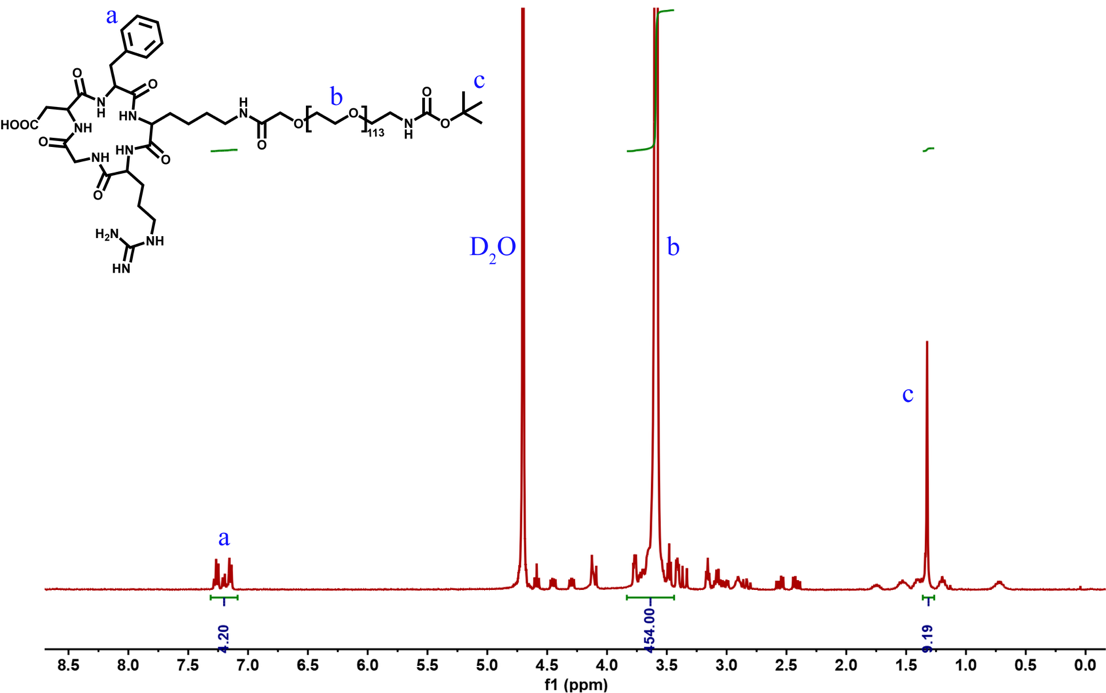


**Figure S7.** ^1^H-NMR spectrum of RGD-PEG-NH-BOC in D_2_O.


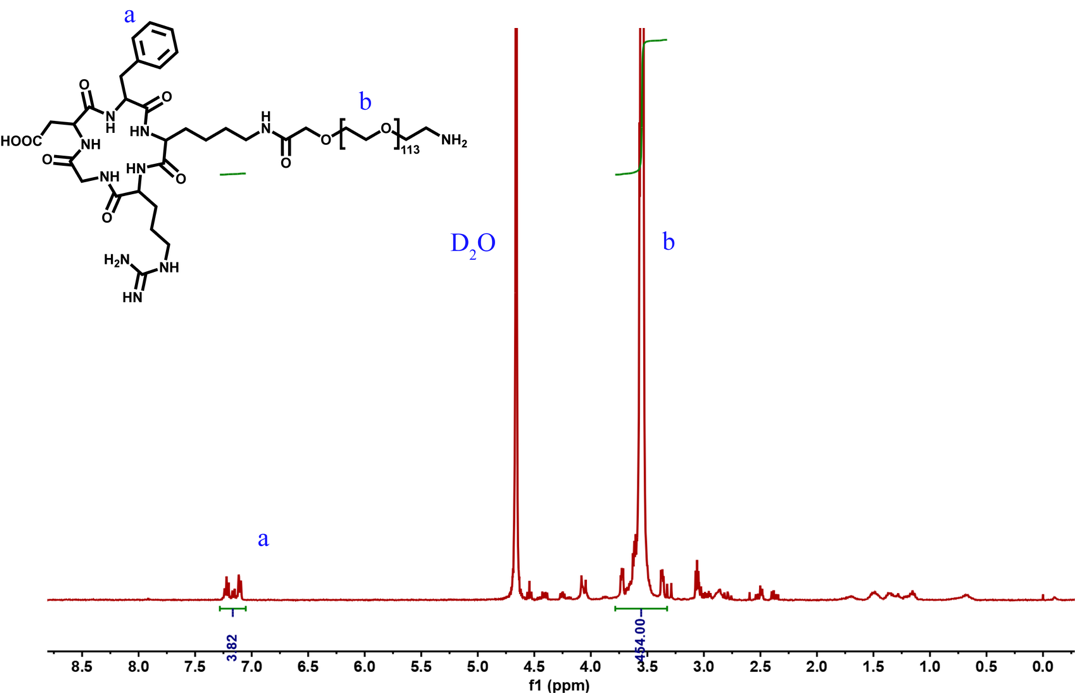


**Figure S8.** ^1^H-NMR spectrum of RGD-PEG-NH_2_ in D_2_O.


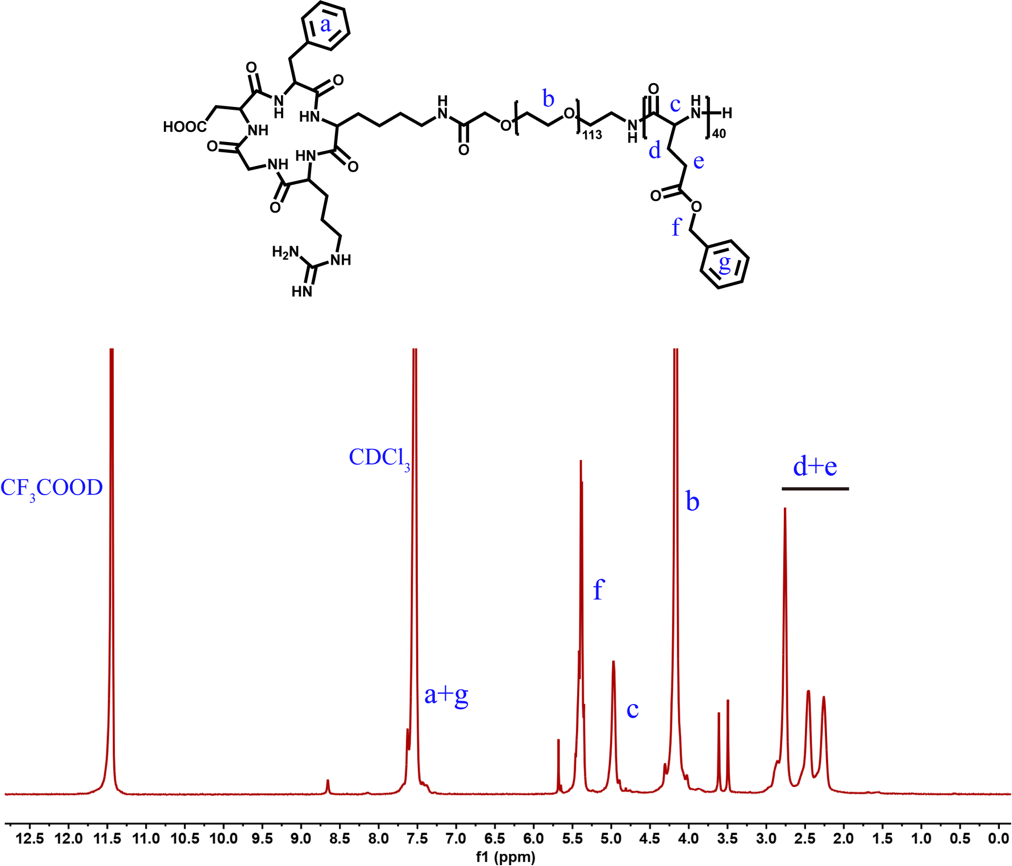


**Figure S9.** ^1^H-NMR spectrum of cRGD-PEG-PGlu (Obzl) in CDCl_3_ and CF_3_COOD (v/v=1:1).


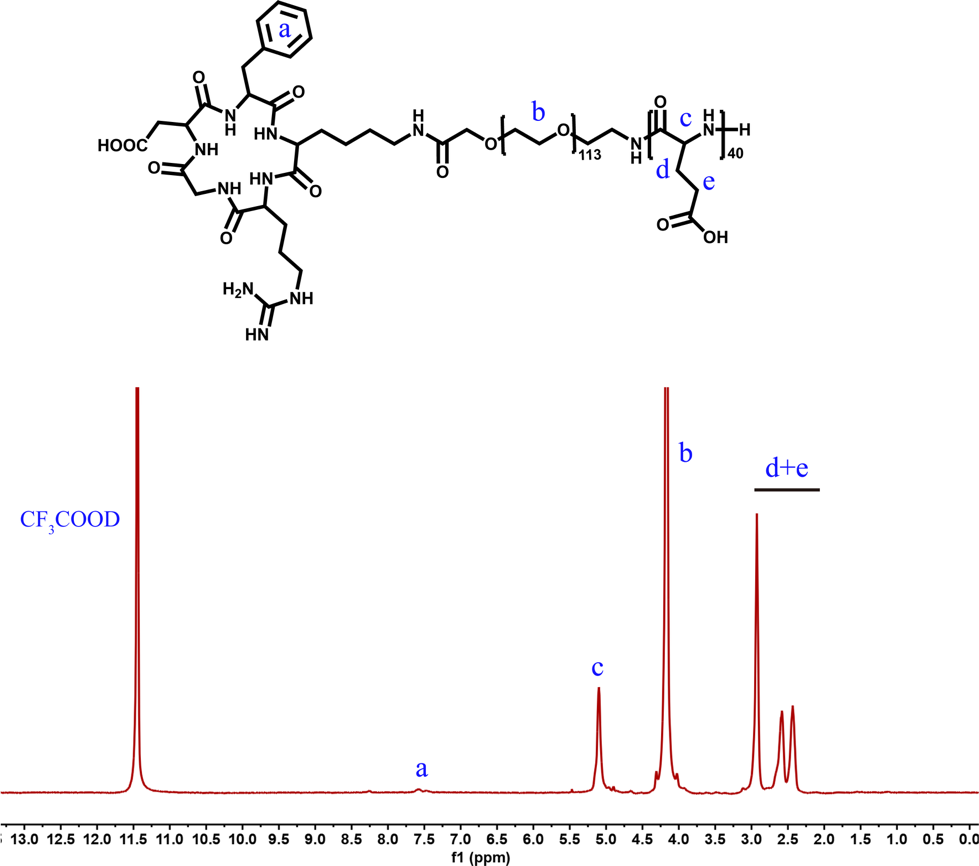


**Figure S10.** ^1^H-NMR spectrum of cRGD-PEG-PGlu in CF_3_COOD.


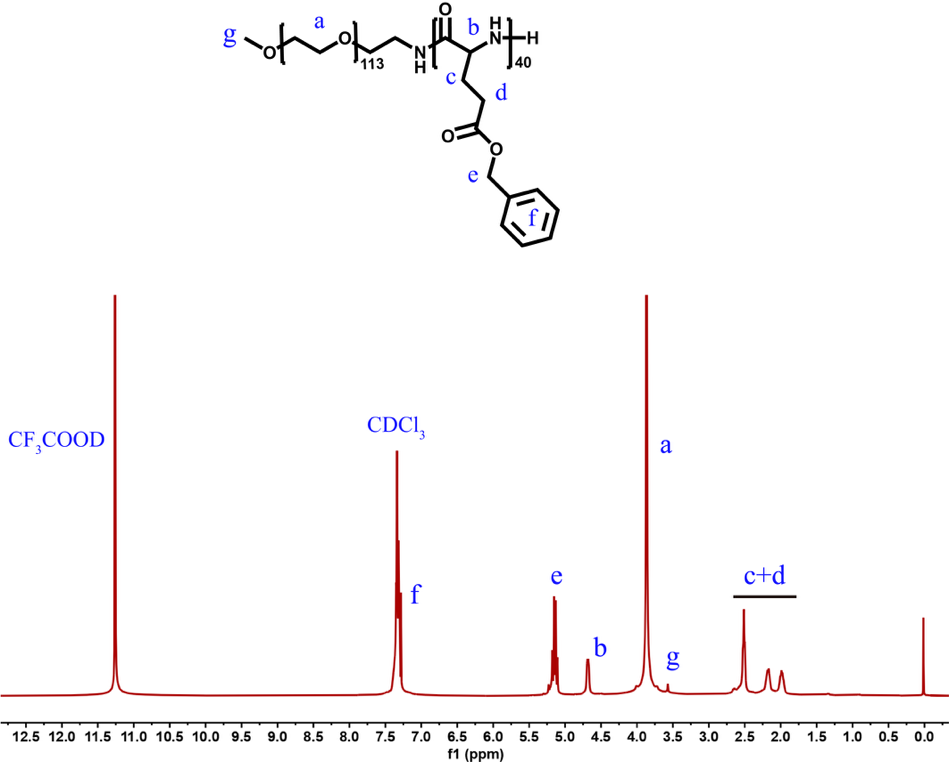


**Figure S11.** ^1^H-NMR spectrum of PEG-PGlu (Obzl) in CDCl_3_ and CF_3_COOD (v/v=1:1).


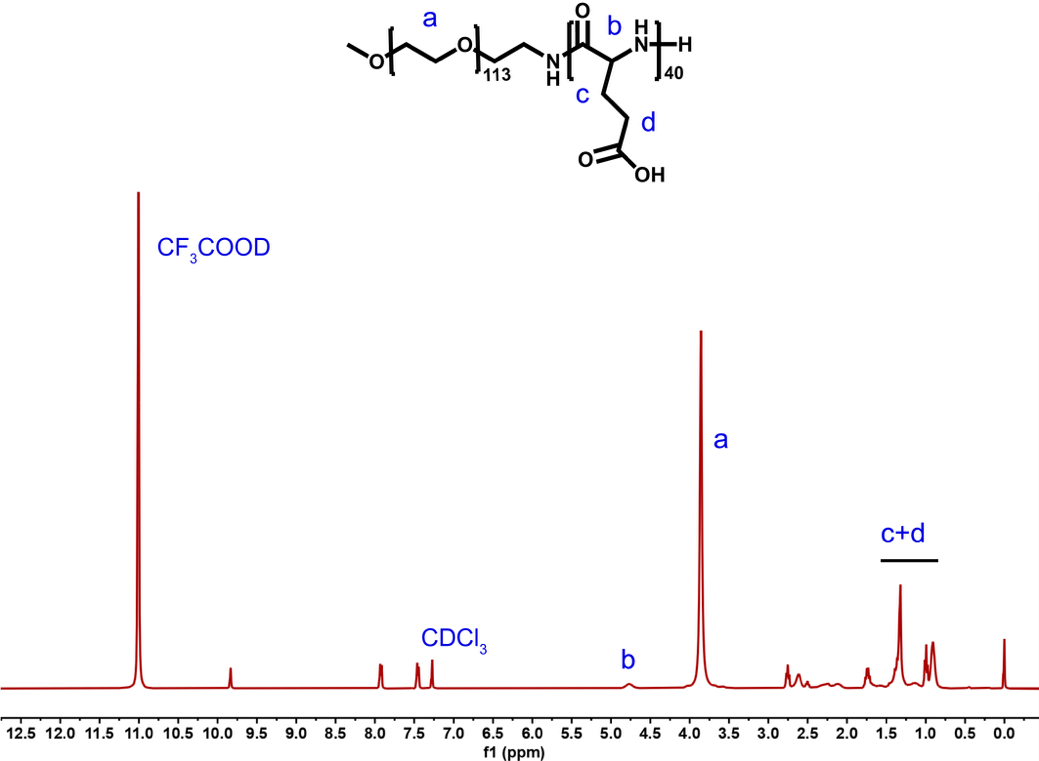


**Figure S12.** ^1^H-NMR spectrum of PEG-PGlu in CDCl_3_ and CF_3_COOD (v/v=1:1).


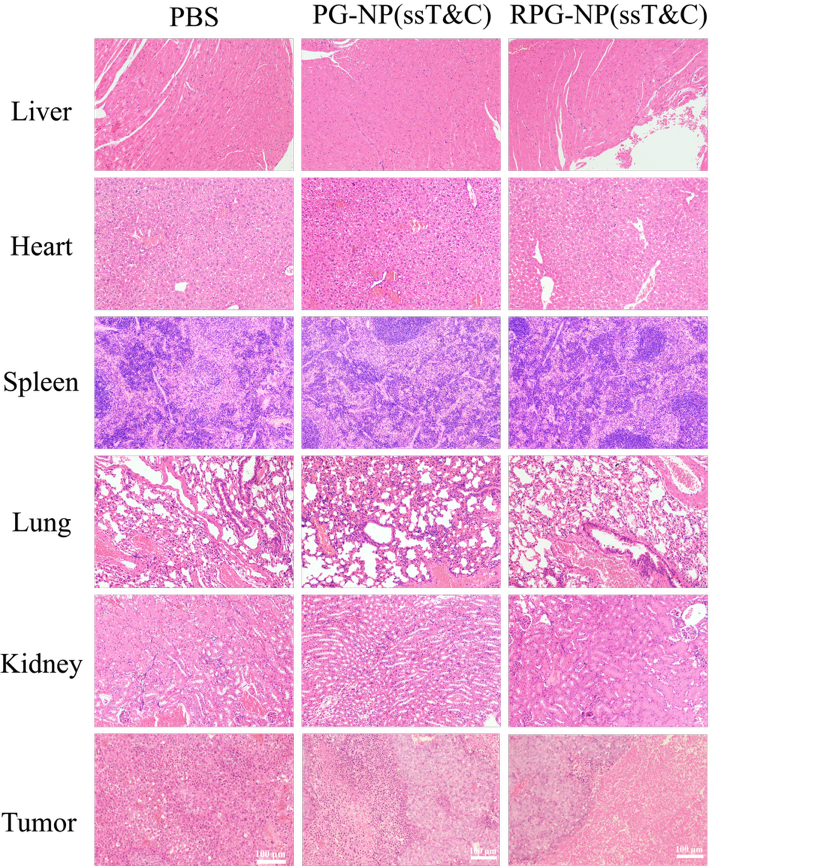


**Figure S13.** H&E staining of tumor sections following designated treatment. (Scale bar: 100 µm).
